# The Role of Myeloperoxidase in Clozapine-Induced Inflammation: A Mechanistic Update for Idiosyncratic Drug-Induced Agranulocytosis

**DOI:** 10.3390/ijms24021243

**Published:** 2023-01-08

**Authors:** Samantha Christine Sernoskie, Alison Jee, Jack Uetrecht

**Affiliations:** 1 Department of Pharmaceutical Sciences, Faculty of Pharmacy, University of Toronto, Toronto, ON M5S 3M2, Canada; 2 Department of Pharmacology and Toxicology, Temerty Faculty of Medicine, University of Toronto, Toronto, ON M5S 1A8, Canada

**Keywords:** myeloperoxidase, MPO, clozapine, inflammation, Sprague Dawley rats, THP-1 macrophages, idiosyncratic drug-induced agranulocytosis

## Abstract

The risk of idiosyncratic drug-induced agranulocytosis (IDIAG) markedly constrains the use of clozapine, a neuroleptic with unparalleled efficacy. Most clozapine patients experience an early inflammatory response, likely a necessary step in IDIAG onset. However, most patients do not progress to IDIAG, presumably because of the requirement of specific human leukocyte antigen (HLA) haplotypes, T cell receptors, and other unknown factors. We established that clozapine activates inflammasomes and that myeloperoxidase bioactivation of clozapine generates neoantigens, but the connection between these early mechanistic events remained unknown and, thus, was the aim of this work. We found that the myeloperoxidase inhibitor PF-1355 attenuated myeloperoxidase activity in phorbol myristate acetate (PMA)-differentiated THP-1 macrophages, and it also attenuated clozapine-induced release of inflammatory mediators (e.g., IL-1β, CXCL1, and C-reactive protein). In vivo, pretreatment of Sprague Dawley rats with PF-1355 significantly attenuated clozapine-induced increases in neutrophil mobilization from the bone marrow to the blood and spleen, as determined using differential blood counts and flow cytometry. Moreover, the clozapine-triggered release of inflammatory mediators (e.g., IL-1β, calprotectin, CXCL1, and α-1-acid glycoprotein) from the liver, spleen, and bone marrow was dampened by myeloperoxidase inhibition. These data support the working hypothesis that oxidation of clozapine to a reactive metabolite by myeloperoxidase is critical for induction of the inflammatory response to clozapine. Ultimately, a better mechanistic understanding of the early events involved in the immune response to clozapine may elucidate ways to prevent IDIAG, enabling safer, more frequent therapeutic use of this and potentially other highly efficacious drugs.

## 1. Introduction

For over 80 years, the risk of idiosyncratic drug-induced agranulocytosis (IDIAG) and other idiosyncratic drug reactions (IDRs) has jeopardized the health of patients [1], the clinical use of highly effective medications [2], and the advancement of novel drug candidates [3,4]. Clozapine, for instance, exhibits superior efficacy in the management of refractory schizophrenia [5,6] and is devoid of extrapyramidal side effects [7], but is significantly underutilized due to the risk of IDIAG [8,9,10]. While safety protocols and mandatory hematological monitoring have greatly reduced the incidence and mortality of IDIAG with clozapine [11,12], limited progress has been made in understanding the mechanistic basis for this reaction. Nevertheless, there is sufficient support for the involvement of an adaptive immune response, believed to be mounted against granulocytes or myeloid precursors [13]. Notably, pharmacogenomic studies have identified positive associations between certain human leukocyte antigen (HLA) haplotypes and the risk of clozapine-induced agranulocytosis [14,15] and in vitro interactions of clozapine with these HLA molecules can generate an adaptive immune response, mediated by CD4^+^ lymphocytes [16]. Clozapine exposure in lymphocytes from patients with a history of IDIAG may also exhibit increased proliferation rates [17], but without HLA genotyping, the clinical relevance is unclear.

An innate immune response is required to induce an adaptive response, and early therapeutic monitoring has indicated that patients usually experience a transient inflammatory response during clozapine initiation [18,19]. Our lab has developed a rat model that recapitulates this early proinflammatory response to clozapine, including the paradoxical neutrophilia and increased proinflammatory mediators observed in patients [20,21]. Using this model, in combination with in vitro studies, we recently demonstrated the requisite of inflammasome signaling during induction of this early immune response to clozapine [22], but the trigger of inflammation was not elucidated.

An absence of myeloid progenitor cells, beginning at the promyelocyte stage, has been shown in the bone marrow of patients with IDIAG [23], and altered neutrophil kinetics, reflective of bone marrow stimulation by granulocyte-colony stimulating factor, have been observed in rats [20] and rabbits [24] acutely treated with clozapine. Interestingly, promyelocytes are the earliest cells to express significant amounts of mature myeloperoxidase [25,26], an enzyme that we hypothesize plays a central role in initiating this early immune response to clozapine [27]. Myeloperoxidase is a heme-containing enzyme, present in mature neutrophils and many myeloid progenitor cells, which is well known for its role in inflammation and host defenses against a wide range of pathogens [28]. Through peroxidase and halogenation cycles, myeloperoxidase catalyzes the formation of reactive species involved in pathogen death, namely free radical molecules and hypohalous acids, respectively. However, myeloperoxidase or myeloperoxidase-generated oxidants also have the capacity to oxidize a number of xenobiotics with a broad range of functional groups, particularly those containing sulfur or nitrogen [29].

**Figure 1:**
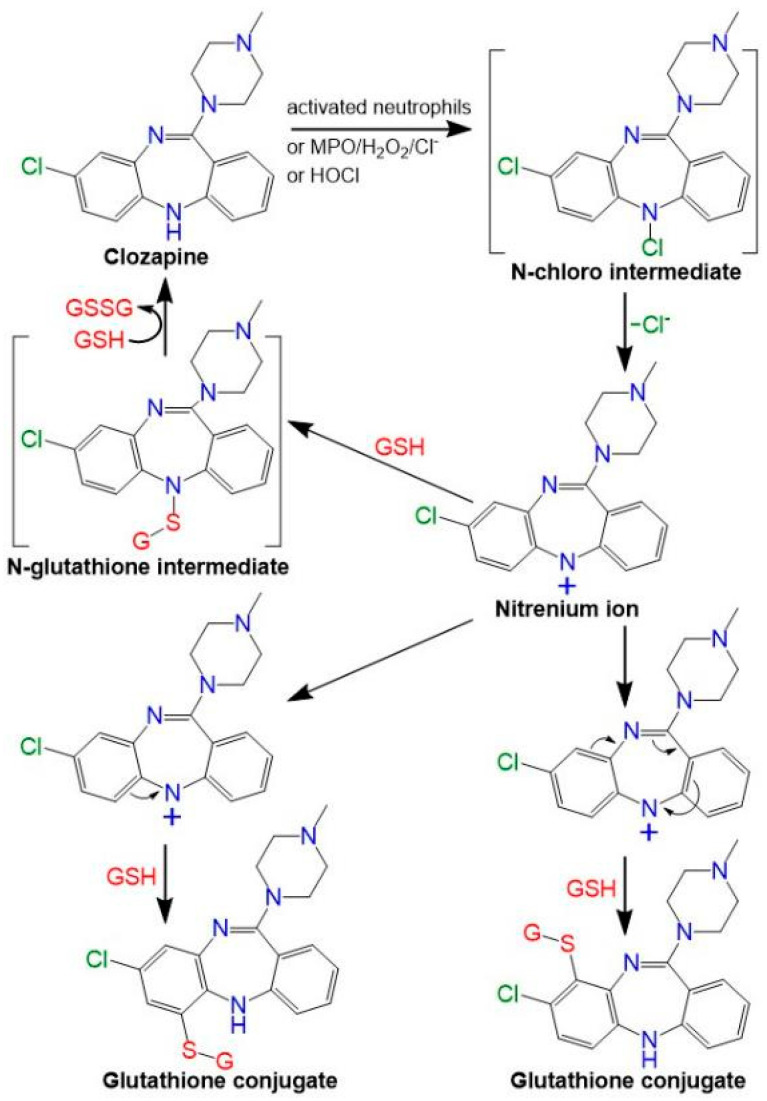
Myeloperoxidase-mediated bioactivation of clozapine [34]. Clozapine can be oxidized by activated neutrophils, oxidant systems containing myeloperoxidase (MPO), hydrogen peroxide (H_2_O_2_), and chloride, or hypochlorous acid to a reactive nitrenium ion that can be trapped by glutathione, resulting in the formation of two major glutathione conjugates [30,31,33]. Glutathione is also likely to react with one or more of the nitrogens; however, these products would be unstable, quickly reacting with another molecule of glutathione to regenerate clozapine and form oxidized glutathione. Due to the delocalization of the positive charge throughout the aromatic system, the nitrenium ion is stable for almost 1 minute in the buffer, much longer than the N-chloro precursor [30]. In the absence of glutathione, the reactive metabolite covalent binds to other peptides and proteins [20,22,27,32,33]. MPO, myeloperoxidase; H_2_O_2_, hydrogen peroxide; HOCl, hypochlorous acid; GSH, reduced glutathione; and GSSG, oxidized glutathione.

Our lab and others have defined the importance of myeloperoxidase in clozapine neoantigen formation [30,31] (Figure 1). We have demonstrated that enzyme systems containing myeloperoxidase generate reactive metabolites of clozapine that covalently modify proteins [30] and that clozapine-treated neutrophils from myeloperoxidase knockout mice exhibit a two-fold decrease in covalent binding compared to wild-type neutrophils [27]. Moreover, clozapine-modified proteins have been detected in vitro (in human neutrophils [32], THP-1 cells [22], HL-60 cells [33]), in vivo (in rats [20,32]), and even in the neutrophils of patients chronically treated with clozapine [32]. However, a link between myeloperoxidase-mediated bioactivation of clozapine and the early immune response to clozapine remains to be defined.

Thus, we hypothesized that the metabolism of clozapine by myeloperoxidase leads to the generation and covalent binding of reactive metabolites to endogenous proteins, causing cellular stress, the release of damage-associated molecular patterns (DAMPs), and the induction of the rapid and transient proinflammatory immune response observed previously. The objectives of this study were to (1) demonstrate that an acute immune response is detectable even at subtherapeutic concentrations of clozapine, as would occur in the initial titration of the clozapine dose in humans, and (2) define the role of myeloperoxidase activation in this proinflammatory response, using a highly selective, 2-thiouracil mechanism-based myeloperoxidase inhibitor, PF-1355 [6-(2,5-dimethoxyphenyl)-3,4-dihydro-4-oxo-2-thioxo-1(2H)-pyrimidineacetamide]. PF-1355 has demonstrated efficacy in reducing inflammation and disease severity in murine models of vasculitis [35] and myocardial infarction and ischemia reperfusion injury [36]. Herein, inhibition of myeloperoxidase significantly dampened the release of DAMPs, the chemotaxis of immune cells, and the induction of inflammation induced by clozapine treatment. These findings expand our understanding of the mechanisms preceding severe IDR onset and indicate that myeloperoxidase activity is necessary for induction of the immune response to clozapine.

## 2. Results

### 2.1. Clozapine Induces Acute Immune Changes at Subtherapeutic Doses In Vivo

#### Clozapine Triggers a Rapid and Dose-Related Inflammatory Immune Response in Rats

**Figure 2:**
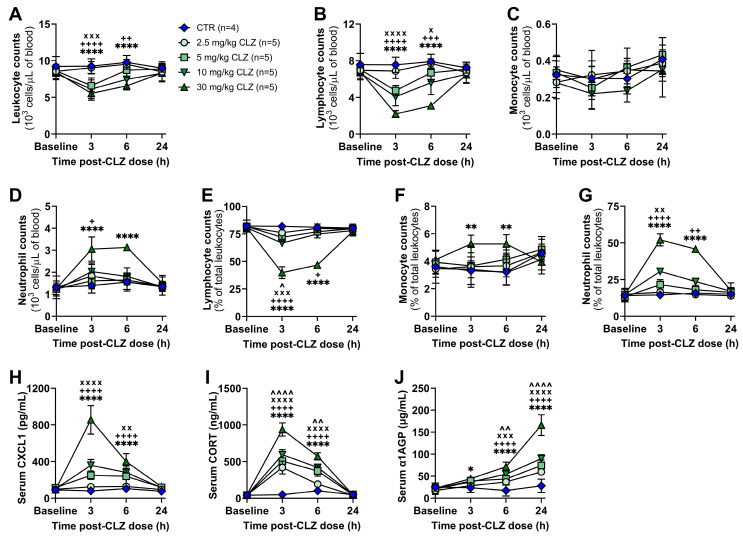
Clozapine triggers dose-related changes in circulating leukocytes and inflammatory mediators in rats. (**A**–**D**) Absolute leukocyte (**A**), lymphocyte (**B**), monocyte (**C**), and neutrophil (**D**) counts, presented as 10^3^ cells/μL of blood, in female Sprague Dawley rats over 24 h following treatment with saline (vehicle control, IP), or clozapine (2.5, 5, 10, or 30 mg/kg, IP). (**E**–**G**) Corresponding lymphocyte (**E**), monocyte (**F**), and neutrophil (**G**) counts, presented as a percentage of total leukocytes. Differential blood counts were determined using a VetScan HM5. (**H**–**J**) Serum concentrations of CXCL1 (**H**), corticosterone (**I**), and α1AGP (**J**) over 24 h post-clozapine administration. Inflammatory mediators were quantified using commercially available ELISA kits. Results are presented as the mean ± SD, and the statistical difference between groups was determined by a repeat-measure two-way ANOVA with the Holm-Sidak’s test for multiple comparisons. CTR, control; CLZ, clozapine; CORT, corticosterone; α1AGP, alpha-1 acid glycoprotein; ^^^, *p* < 0.05; ^^^^, *p* < 0.01; ^^^^^^, *p* < 0.0001 (2.5 mg/kg clozapine vs. control); ^x^, *p* < 0.05; ^xx^, *p* < 0.01; ^xxx^, *p* < 0.001; ^xxxx^, *p* < 0.0001 (5 mg/kg clozapine vs. control); ^+^, *p* < 0.05; ^++^, *p* < 0.01; ^+++^, *p* < 0.001; ^++++^, *p* < 0.0001 (10 mg/kg clozapine vs. control); **, *p* < 0.01; ****, *p* < 0.0001 (30 mg/kg clozapine vs. control).

Previously, our lab demonstrated that the treatment of female Sprague Dawley rats with a dose of clozapine that approximates therapeutic drug levels results in a rapid and transient proinflammatory response [20,22], with features similar to those reported in patients during the first month of clozapine treatment [18,19,37,38,39]. However, to reduce the risk of seizures, hypotension, and other adverse events, patients are typically titrated to their final clozapine dose over several weeks [40,41,42], with initial drug exposures at subtherapeutic concentrations. Unfortunately, no patient hematology data is currently available for time points earlier than one week of continuous treatment at these lower doses. Thus, to expand the potential clinical relevance of our model and the mechanisms reported, the immune responses to several lower doses of clozapine, representing exposure levels of new clozapine users, were investigated. Specifically, changes in circulating leukocyte populations and inflammatory mediators were assessed over 24 h following a single clozapine dose (2.5, 5, 10, or 30 mg/kg) in rats. From 3 to 6 h, a significant reduction in absolute leukocyte counts was detectable with 5, 10, and 30 mg/kg doses (*p* < 0.0001—*p* < 0.01; Figure 2A), which was caused by a decrease in lymphocytes that was detectable at all doses (*p* < 0.05—*p* < 0.0001; Figure 2B,E). Over the same time, an increase in circulating neutrophils was observed for the three highest doses tested (Figure 2D,G), with lymphocyte and neutrophil counts returning to baseline by 24 h. Monocyte counts were unchanged at all doses (Figure 2C,F). 

Corresponding to the increase in neutrophils, serum levels of the neutrophil chemokine CXCL1 were also elevated from 3 to 6 h with 5—30 mg/kg doses (*p* < 0.01—*p* < 0.0001; Figure 2H), with spleen and bone marrow CXCL1 levels increasing at 24 h (Supplemental Figure S1B,C). Corticosterone, the major circulating glucocorticoid in rodents [43], was increased in the serum from 3 to 6 h at all doses (*p* < 0.01—*p* < 0.0001; Figure 2I). This increase is likely involved in mediating the acute lymphopenia observed because corticosteroid administration can result in a profound yet transient decrease in peripheral lymphocytes within hours, similar to the pattern observed here [44,45,46]. This may also explain the slight decrease in organ weights observed across all doses at 24 h (Supplemental Figure S2). Alpha-1 acid glycoprotein (α1AGP), an acute-phase protein that, in rats, has comparable function to C-reactive protein (CRP) in humans, was significantly elevated at all doses by 6 h in the serum (*p* < 0.05—*p* < 0.0001; Figure 2J) and was also increased in the liver, spleen, and bone marrow at 24 h (Supplemental Figure S1G–I). Together, these data suggest that, even at subtherapeutic clozapine exposure levels, patients may experience an acute inflammatory response within the first hours to days of treatment, warranting early hematological monitoring in new clozapine users to validate these preclinical findings.

### 2.2. Myeloperoxidase Inhibition Attenuates Clozapine-Induced Inflammatory Mediator Release In Vitro

#### 2.2.1. Clozapine-Induced Increases in Myeloperoxidase Activity Are Dampened by Myeloperoxidase Inhibition, without Affecting Viability

Having demonstrated that clozapine can induce an immune response in rats at doses well below therapeutic levels, we returned to our major question: what role does myeloperoxidase play in initiating this immune response? Myeloperoxidase has an established role in clozapine neoantigen formation [30,31] and ex vivo clozapine covalent binding, as demonstrated using neutrophils from myeloperoxidase knockout mice [27]. Mice, however, cannot tolerate therapeutic clozapine concentrations and experience extreme sedation [27], preventing the in vivo investigation of the role of myeloperoxidase using these knockout mice. In the absence of a myeloperoxidase knockout rat model, PF-1355, a highly selective, 2-thiouracil mechanism-based myeloperoxidase inhibitor with high preclinical efficacy [35,36], was employed to determine the necessity of myeloperoxidase in the induction of the proinflammatory response to clozapine. The pharmacokinetics of PF-1355 have been characterized in several preclinical models, including rats, and PF-1355 was demonstrated to exhibit a reduction in inflammation comparable to that observed in myeloperoxidase knockout mice in a model of immune complex vasculitis [35]. Nevertheless, to perform our own validation, the inhibitory effects of PF-1355 on myeloperoxidase were examined in phorbol myristate acetate (PMA)-differentiated THP-1 macrophages, our simplified model to investigate myeloid cell-mediated inflammation in vitro, and in neutrophils isolated from rats treated in vivo.

**Figure 3:**
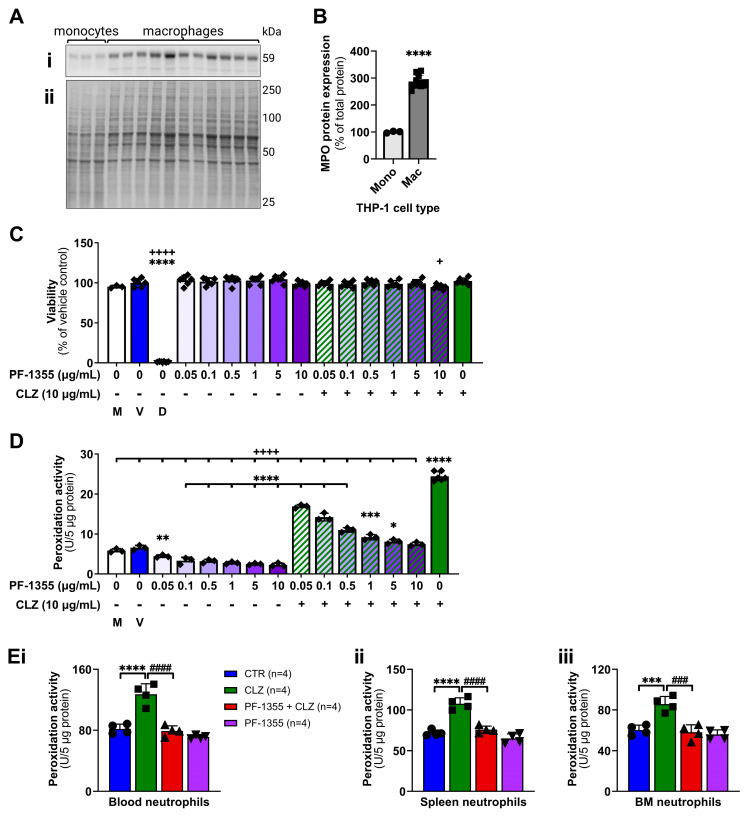
Clozapine increases myeloperoxidase activity in THP-1 macrophages and rat neutrophils. (**A**,**B**) Immunoblot analysis to detect myeloperoxidase protein expression in THP-1 cells. Protein (10 μg) from THP-1 monocytes or PMA-differentiated macrophages was run on 10% precast gels, transferred to the nitrocellulose membrane, and stained for total protein (Ai) and myeloperoxidase (Aii), prior to densitometry analysis (**B**). Myeloperoxidase band density was normalized to total protein and data are presented as a percentage of monocyte myeloperoxidase expression. (**C**) Cell viability of THP-1 macrophages after 24 h of incubation with media, vehicle control, 10% DMSO, clozapine (10 μg/mL), PF-1355 (0.05–10 μg/mL), or clozapine with PF-1355. Viability was determined using a CCK-8 assay and data are presented as a percent of vehicle controls. (**D**,**E**) Myeloperoxidase peroxidation activity in THP-1 macrophages after 3 h of incubation with media, vehicle control, 10% DMSO, clozapine (10 μg/mL), PF-1355 (0.05–10 μg/mL), or coincubation with clozapine and PF-1355 (**D**), or in blood (**Ei**), spleen (**ii**), or bone marrow (**iii**) neutrophils isolated from female Sprague Dawley rats at 3 h following treatment with vehicle controls, PF-1355 (100 mg/kg, PO), clozapine (30 mg/kg, IP), or PF-1355 and clozapine. Enzyme activity was determined using fluorescent substrate-based assays and data are presented as enzyme units per protein. Results are presented as the mean ± SD and the statistical difference between groups was determined by t-test or one-way ANOVA with the Holm-Sidak’s test for multiple comparisons. MPO, myeloperoxidase; Mono, monocytes; Mac, macrophages; M, media alone control; V, vehicle control; D, 10% DMSO positive control; CLZ, clozapine; BM, bone marrow; *, *p* < 0.05; **, *p* < 0.01; ***, *p* < 0.001; ****, *p* < 0.0001 (treatment group vs. control); ^+^, *p* < 0.05; ^++++^, *p* < 0.0001 (treatment group vs. clozapine); ^###^, *p* < 0.001; ^####^, *p* < 0.0001 (PF-1355 + clozapine vs. clozapine).

Myeloperoxidase is abundantly expressed in neutrophils and is also detectable in monocytes and certain tissue macrophages [47,48]. However, there have been conflicting reports of whether THP-1 cells express [49] or do not express myeloperoxidase [50]. Thus, the protein expression of myeloperoxidase was first verified using western blotting (Figure 3A,B) where, indeed, myeloperoxidase was detected at low levels in THP-1 monocytes and was upregulated in PMA-differentiated THP-1 macrophages (*p* < 0.0001). Next, the viability of THP-1 macrophages was assessed following a 24 h incubation with increasing concentrations of PF-1355 (0.05–10 μg/mL) ± clozapine (10 μg/mL) to ensure that PF-1355 did not cause cytotoxicity alone or in combination with clozapine (Figure 3C). No differences in viability were observed compared to vehicle control. The effects of clozapine and PF-1355 on myeloperoxidase enzyme activity were then characterized using both THP-1 macrophages (Figure 3D) and neutrophils isolated from rats treated with clozapine (30 mg/kg, IP) and PF-1355 (100 mg/kg, PO) in vivo (Figure 3E). Following a 3 h incubation, clozapine alone caused significant increases in peroxidation activity in THP-1 macrophages (*p* < 0.0001), consistent with clozapine-mediated increases in myeloperoxidase-catalyzed enzymatic activity that have been demonstrated by ultraviolet–vis spectrophotometry [51]. Incubation with increasing concentrations of PF-1355 significantly attenuated clozapine-mediated increases in peroxidation activity in a dose-dependent manner (*p* < 0.05—*p* < 0.0001), with all concentrations of PF-1355 decreasing peroxidation activity compared to vehicle control (*p* < 0.01—*p* < 0.0001). Neutrophils isolated from the blood, spleen, and bone marrow of rats at 3 h post-clozapine treatment demonstrated a similar pattern, whereby clozapine alone significantly increased peroxidation activity (*p* < 0.001—*p* < 0.0001) and pretreatment with PF-1355 attenuated this increase (*p* < 0.001—*p* < 0.0001).

#### 2.2.2. Clozapine-Induced Increases in Inflammatory Mediator Release Are Dampened by Myeloperoxidase Inhibition In Vitro

**Figure 4:**
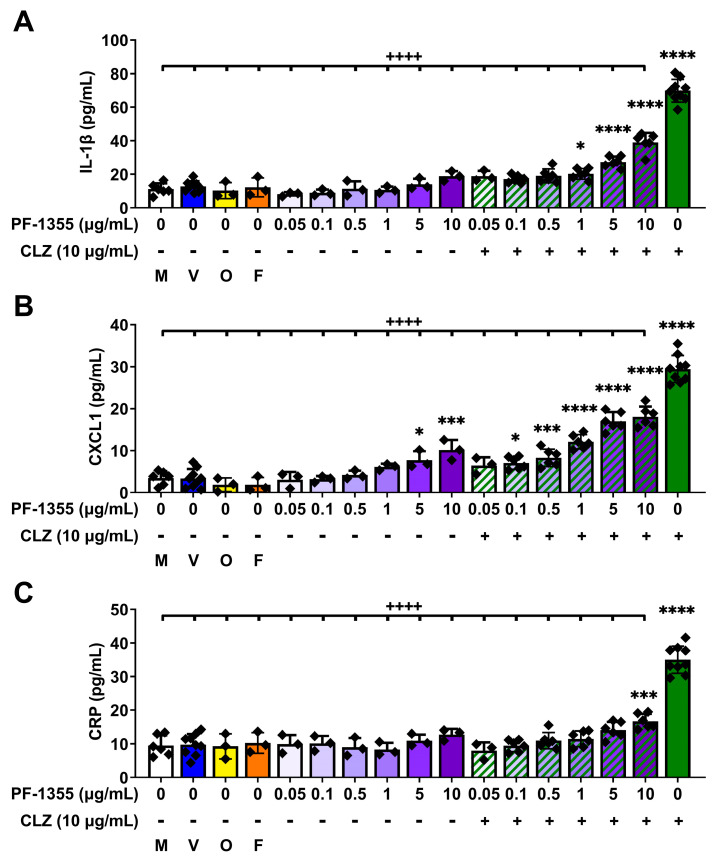
PF-1355 attenuates clozapine-induced increases in inflammatory mediator release in THP-1 macrophages. (**A**–**C**) Supernatant concentrations of IL-1β (**A**), CXCL1 (**B**), or CRP (**C**) in THP-1- macrophages after 24 h of incubation with media, vehicle control, olanzapine (10 μg/mL), fluperlapine (10 μg/mL), clozapine (10 μg/mL), PF-1355 (0.05–10 μg/mL), or coincubation with clozapine and PF-1355 (1–10 μg/mL). Inflammatory mediators were quantified using commercially available ELISAs. Results are presented as the mean ± SD and statistical difference between groups was determined by a one-way ANOVA with the Holm-Sidak’s test for multiple comparisons. M, media alone control; V, vehicle control; O, olanzapine (negative control); F, fluperlapine (negative control); CLZ, clozapine; *, *p* < 0.05; ***, *p* < 0.001; ****, *p* < 0.0001 (treatment group vs. control); ^++++^, *p* < 0.0001 (treatment group vs. clozapine).

Prior studies from our lab and collaborators have highlighted that inflammasome activation and release of IL-1β occur in vitro with a multitude of IDR-associated drugs and/or their metabolites [52,53,54,55,56,57], including clozapine [22]. IL-1β is considered to be a master regulator of inflammation and has widespread effects on the inflammatory response [58,59]. Production of IL-1β and its downstream mediator CXCL1 were significantly attenuated by myeloperoxidase inhibition after 24 h in clozapine-treated THP-1 macrophages (*p* < 0.0001; Figure 4A,B), indicating that myeloperoxidase plays a role in cytokine release upstream of inflammasome activation. Moreover, clozapine was found to increase CRP release in THP-1 macrophages; an effect that was also blocked by PF-1355 (*p* < 0.0001; Figure 4C). Interestingly, treatment with the highest concentrations of PF-1355 alone caused slight increases in CXCL1 (*p* < 0.05—*p* < 0.001; Figure 4B). Since PF-1355 is a mechanism-based inhibitor, it is possible that high levels of PF-1355 covalently bound to myeloperoxidase can cause cell stress and an inflammatory response, much like what occurs with clozapine. However, the proinflammatory effects exerted by clozapine were significantly greater than those of PF-1355 and did not occur synergistically, as coincubation of PF-1355 was effective in dampening both the increases in myeloperoxidase activity and subsequent release of inflammatory mediators induced by clozapine. Together, these in vitro findings support a role for myeloperoxidase-mediated clozapine bioactivation in the mechanism of the early proinflammatory response induced by clozapine, which occurs upstream of inflammasome activation.

### 2.3. Myeloperoxidase Inhibition Attenuates Clozapine-Induced Inflammation In Vivo

#### 2.3.1. Clozapine-Induced Increases in Neutrophil Mobilization Are Dampened by Myeloperoxidase Inhibition

**Figure 5:**
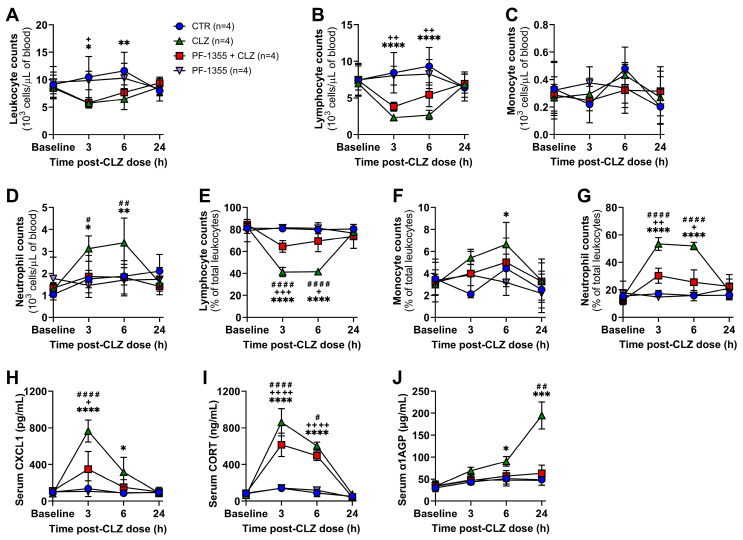
PF-1355 attenuates clozapine-induced increases in circulating neutrophil and inflammatory mediators in rats. (**A**–**D**) Absolute leukocyte (**A**), lymphocyte (**B**), monocyte (**C**), and neutrophil (**D**) counts, presented as 10^3^ cells/μL of blood, in female Sprague Dawley rats over 24 h following treatment with vehicle controls, PF-1355 (100 mg/kg, PO), clozapine (30 mg/kg, IP), or PF-1355 and clozapine. (**E**–**G**) Corresponding lymphocyte (**E**), monocyte (**F**), and neutrophil (**G**) counts, presented as a percentage of total leukocytes. Differential blood counts were determined using a VetScan HM5. (**H**–**J**) Serum concentrations of CXCL1 (**H**), corticosterone (**I**), and α1AGP (**J**) over 24 h post-clozapine administration. Inflammatory mediators were quantified using commercially available ELISA kits. Results are presented as the mean ± SD and the statistical difference between groups was determined by a repeat-measure two-way ANOVA with the Holm-Sidak’s test for multiple comparisons. CTR, control; CLZ, clozapine; *, *p* < 0.05; **, *p* < 0.01; ***, *p* < 0.001; ****, *p* < 0.0001 (clozapine vs. control); ^+^, *p* < 0.05; ^++^, *p* < 0.01; ^+++^, *p* < 0.001; ^++++^, *p* < 0.0001 (PF-1355 + clozapine vs. control); ^#^, *p* < 0.05; ^##^, *p* < 0.01; ^####^, *p* < 0.0001 (PF-1355 + clozapine vs. clozapine).

To define the role of myeloperoxidase during induction of the immune response to clozapine in our rodent model, rats were pretreated with PF-1355 1 h prior to clozapine administration, and immune parameters were evaluated up to 24 h post-clozapine dose. While myeloperoxidase inhibition did not substantially affect total leukocyte counts compared to treatment with clozapine alone (Figure 5A), clozapine-induced decreases in lymphocyte counts were slightly attenuated by PF-1355 from 3 to 6 h (*p* < 0.0001; Figure 5B,E). Additionally, inhibition of myeloperoxidase significantly damped clozapine-mediated increases in neutrophil counts from 3 to 6 h (*p* < 0.05—*p* < 0.0001; Figure 5D,G), without clearly affecting monocyte counts (Figure 5C,F). PF-1355 pre-treatment was also found to greatly dampen clozapine-mediated increases in CXCL1 at 3 h (*p* < 0.0001; Figure 5H), corticosterone from 3 to 6 h (*p* < 0.05—*p* < 0.0001; Figure 5I), and α1AGP at 24 h (*p* < 0.01; Figure 5J). These data indicate that myeloperoxidase is necessary for the inflammatory immune response to clozapine and that the effects of this response can be clearly demonstrated in peripheral blood samples.

#### 2.3.2. Clozapine-Induced Neutrophil Chemotaxis Is Dampened by Myeloperoxidase Inhibition

**Figure 6:**
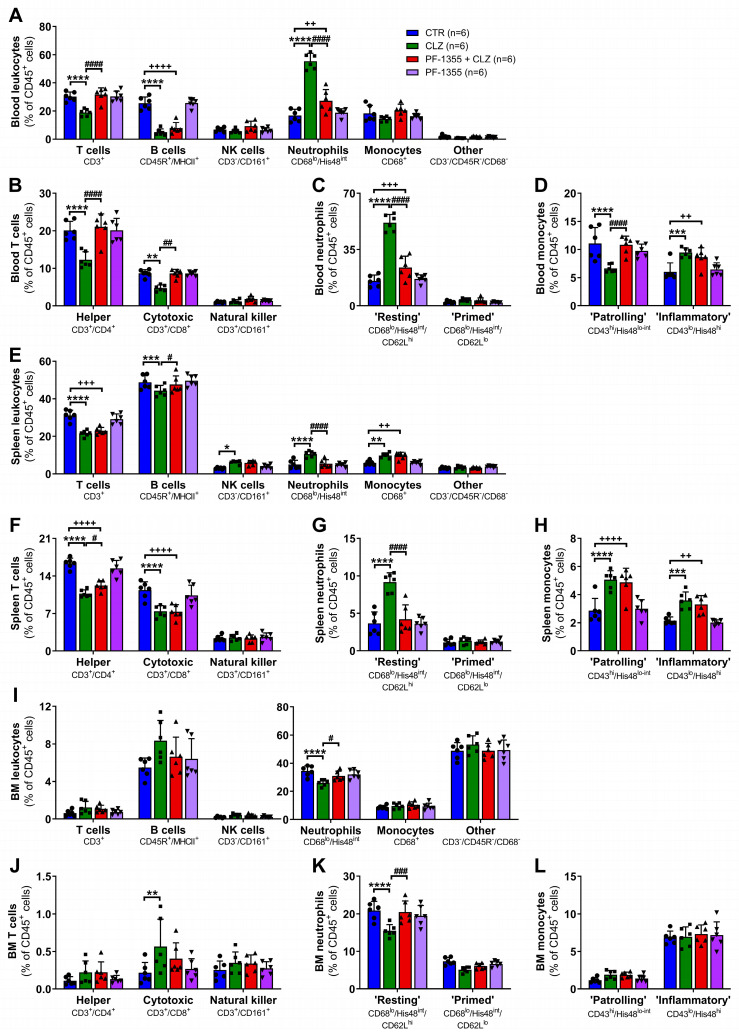
PF-1355 pretreatment attenuates clozapine-induced changes in neutrophil mobilization and other immune subsets in rats. Flow cytometric phenotyping of basic immune cell populations (**A**,**E**,**I**), and T lymphocyte (**B**,**F**,**J**), neutrophil (**C**,**G**,**K**), and monocyte (**D**,**H**,**L**) subsets in the blood (**A**–**D**), spleen (**E**–**H**), and bone marrow (**I**–**L**) presented as percentage of live, CD45^+^ leukocytes in female Sprague Dawley rats at 3 h following treatment with vehicle controls, PF-1355 (100 mg/kg, PO), clozapine (30 mg/kg, IP), or PF-1355 and clozapine. Cell suspensions were stained for cell surface markers and viability, fixed, permeabilized, and stained for intracellular markers, prior to flow cytometric analysis. Results are presented as the mean ± SD and the statistical difference between groups was determined by a two-way ANOVA with the Holm-Sidak’s test for multiple comparisons. CTR, control; CLZ, clozapine; NK, natural killer; BM, bone marrow; *, *p* < 0.05; **, *p* < 0.01; ***, *p* < 0.001; ****, *p* < 0.0001 (clozapine vs. control); ^+^, *p* < 0.05; ^++^, *p* < 0.01; ^+++^, *p* < 0.001; ^++++^, *p* < 0.0001 (PF-1355 + clozapine vs. control); ^#^, *p* < 0.05; ^##^, *p* < 0.01; ^###^, *p* < 0.001; ^####^, *p* < 0.0001 (PF-1355 + clozapine vs. clozapine).

Using a rat-specific antibody panel adapted from Barnett-Vanes et al. [60], flow cytometric phenotyping of leukocytes was conducted at 3 h post-clozapine to better characterize the cell populations involved during the peak of this early immune response. In accordance with the changes observed using differential blood counts, clozapine treatment significantly increased blood neutrophils (*p* < 0.0001; Figure 6A) and decreased both T and B lymphocytes (*p* < 0.0001). Interestingly, myeloperoxidase inhibition with PF-1355 attenuated changes in both neutrophil and T lymphocyte populations (*p* < 0.0001) but did not rescue decreases in B lymphocytes. Specific cell subsets were also characterized, and it was found that clozapine caused decreases in circulating CD4^+^ helper (*p* < 0.0001, Figure 6B) and CD8^+^ cytotoxic (*p* < 0.01) T lymphocytes, both of which were rescued by myeloperoxidase inhibition (*p* < 0.01—*p* < 0.0001). Conversely, clozapine selectively increased CD62L^hi^ mature resting neutrophils (*p* < 0.0001; Figure 6C), an observation that is consistent with other models of acute non-pathogenic inflammation [61] and has also been observed following 10 days of repeat clozapine treatment in our rat model [20]. Again, PF-1355 pretreatment abrogated this increase. Although no significant change in total monocytes was observed, clozapine did trigger a shift from an M2 patrolling/non-classical phenotype (His48^lo^/CD43^hi^ monocytes, analogous to Ly6C^lo^ monocytes in mice [62]) to an M1 inflammatory/classical phenotype (His48^hi^/CD43^lo^ monocytes, analogous to Ly6C^hi^ monocytes in mice [62]) (*p* < 0.001—*p* < 0.0001; Figure 6D), but myeloperoxidase inhibition only dampened the reduction in the M2 subset (*p* < 0.0001). Clozapine caused a similar pattern of leukocyte changes in the spleen (Figure 6E-H), with attenuation of the increase in neutrophils noted in the PF-1355 pretreated group (*p* < 0.0001; Figure 6G). Few changes were observed in the mature immune cell populations of the bone marrow with clozapine treatment (Figure 6I–L), aside from a decrease in resting neutrophils (*p* < 0.0001; Figure 6I,K) that was dampened by myeloperoxidase inhibition (*p* < 0.05—*p* < 0.001). Taken together, these results illustrate that clozapine causes an increase in mature neutrophil release from the bone marrow and an increase in neutrophil chemotaxis through the blood and spleen, without apparent neutrophil activation. Clozapine also triggers a decrease in T and B lymphocytes; however, myeloperoxidase activity appears to predominantly be involved in the mobilization of neutrophils and the phenotypic shift in monocytes.

#### 2.3.3. Clozapine-Induced Organ-Specific DAMP and Inflammatory Mediator Release Are Dampened by Myeloperoxidase Inhibition

**Figure 7:**
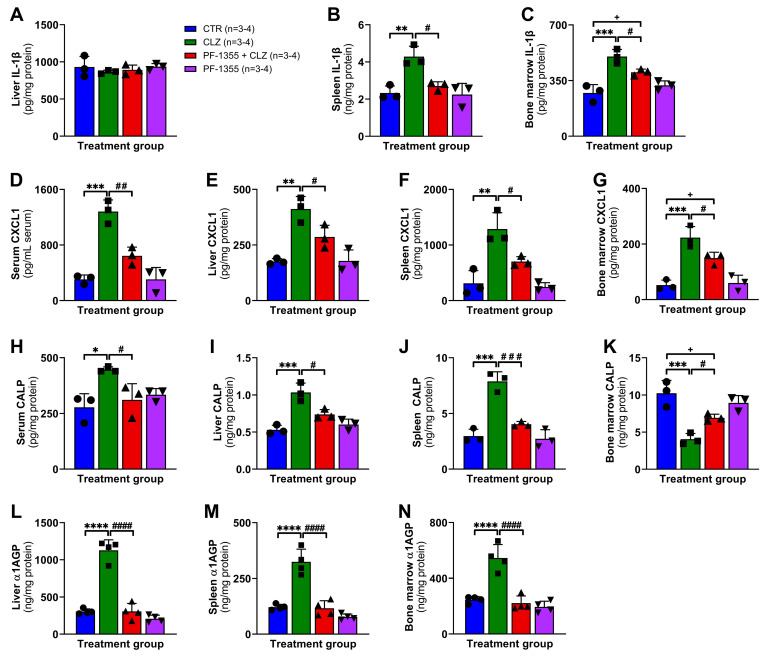
PF-1355 pretreatment attenuates the clozapine-induced release of DAMPs and inflammatory mediators in rats**.** (**A**–**K**) Concentrations of IL-1β (**A**–**C**), CXCL1 (**D**–**G**), and calprotectin (**H**–**K**) in the liver (**A**,**E**,**I**), spleen (**B**,**F**,**J**), bone marrow (**C**,**G**,**K**), and serum (**D**,**H**) in female Sprague Dawley rats at 3 h following treatment with vehicle (control) or clozapine (2.5, 5, 10, or 30 mg/kg, IP). Concentrations of α1AGP (**L**–**N**) in the liver (**L**), spleen (**M**), and bone marrow (**N**) at 24 h post-clozapine administration. Inflammatory mediators were quantified using commercially available ELISA kits. Results are presented as the mean ± SD and the statistical difference between groups was determined by a one-way ANOVA with the Holm-Sidak’s test for multiple comparisons. CTR, control; CLZ, clozapine; CALP, calprotectin; α1AGP, alpha-1 acid glycoprotein; *, *p* < 0.05; **, *p* < 0.01; ***, *p* < 0.001; ****, *p* < 0.0001 (clozapine vs. control); ^+^, *p* < 0.05 (PF-1355 + clozapine vs. control); ^#^, *p* < 0.05; ^##^, *p* < 0.01; ^###^, *p* < 0.001; ^####^, *p* < 0.0001 (PF-1355 + clozapine vs. clozapine).

Lastly, to test the hypothesis that the immune response to clozapine is triggered by cellular damage, specifically the release of DAMPs following myeloperoxidase-mediated bioactivation of clozapine, tissue sources of DAMPs and other inflammatory mediators were investigated. Having found that clozapine-induced release of IL-1β, the main DAMP generated during inflammasome activation, was attenuated by myeloperoxidase inhibition in vitro (Figure 4A), this was also examined in vivo. Additionally, the DAMP calprotectin, which is also known as the heterodimer of S100A8/A9, was studied as it accounts for up to half of the cytoplasmic protein content in neutrophils and is a well-established biomarker for several inflammatory diseases [63,64,65,66]. Calprotectin has diverse functions in the inflammatory response, including regulating inflammatory cell chemotaxis, adhesion, and activation [67]. At 3 h, clozapine caused significant increases in IL-1β levels in the spleen (*p* < 0.01; Figure 7B) and bone marrow (*p* < 0.001; Figure 7C) that were significantly dampened by PF-1355 pre-treatment (*p* < 0.05). The downstream release of CXCL1 was elevated by clozapine across tissues examined (*p* < 0.01—*p* < 0.001; Figure 7D–G) and was also attenuated by myeloperoxidase inhibition (*p* < 0.05—*p* < 0.01). In response to clozapine, calprotectin levels were slightly elevated in the serum (*p* < 0.05; Figure 7H) and were more robustly elevated in the liver and spleen (*p* < 0.001; Figure 7I–J) but were decreased in the bone marrow (*p* < 0.001; Figure 7K). This was an unexpected result, but the changes in calprotectin observed parallel the mobilization of neutrophils out of the bone marrow and into the circulation, and it is reasonable to conclude that decreased bone marrow calprotectin represents a loss of neutrophils with high intracellular concentrations of DAMP. Importantly, these changes were also attenuated by the inhibition of myeloperoxidase (*p* < 0.05—*p* < 0.001). Lastly, in addition to the clozapine-mediated increases in serum α1AGP that were attenuated by myeloperoxidase inhibition at 24 h (Figure 5J), clozapine-mediated release of α1AGP from the liver, spleen, and bone marrow was also prevented by PF-1355 pre-treatment (*p* < 0.0001; Figure 7L–N). Overall, these findings support our working model of clozapine-induced immune activation, whereby myeloperoxidase bioactivation of clozapine causes cell stress, the release of DAMPs and other inflammatory mediators, and the induction of a robust proinflammatory response.

## 3. Discussion

Clozapine remains an important antischizophrenic treatment option, but the risk of IDIAG severely limits its use [68] and our understanding of the etiology of IDIAG remains incomplete [19]. Over the past 30 years, our lab has investigated the metabolic and immune components of this reaction [69], focusing on the acute mechanistic events. Previously, we and others defined the importance of myeloperoxidase in clozapine neoantigen formation [30,31]. Most recently, we demonstrated the requisite of inflammasome signaling in induction of the immune response to clozapine [22], but how clozapine bioactivation and this immune activation were linked remained unclear. In the present study, we explored the relevance of our rodent model in the context of new clozapine users receiving subtherapeutic doses of clozapine prior to characterizing the role of myeloperoxidase during the early immune response to clozapine using both in vitro and in vivo approaches.

Herein, we have provided justification for acute hematological monitoring in patients commencing clozapine treatment, as clozapine doses of less than one-tenth of what correspond to therapeutic blood levels in our rat model still induced a detectable inflammatory response (Figure 2). If certain patients exhibit a more robust proinflammatory response early in treatment, it would be interesting to determine if they are at an increased risk of progression to IDIAG later in treatment. In support of this possibility, our lab previously demonstrated that in a D-penicillamine-induced autoimmune disease model, treated rats that developed higher serum cytokine levels also developed more severe autoimmunity and became more ill compared to non-responders [70].

**Figure 8:**
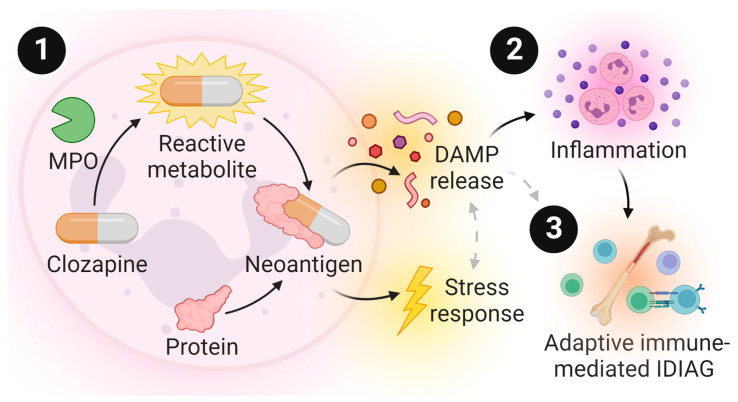
An updated model linking clozapine bioactivation and clozapine-induced inflammation in idiosyncratic drug-induced agranulocytosis (IDIAG). (1) Clozapine is oxidized by myeloperoxidase (MPO)-expressing cells to a reactive nitrenium ion that covalently modifies endogenous proteins, generating neoantigens. Depending on the essentiality and the role of the proteins modified, this causes cellular dysfunction, the release of damage-associated molecular patterns (DAMPs; including S100 and drug-modified proteins), along with other proinflammatory mediators, and activation of a stress response, propagated by cortisol/corticosterone. The stress response may also directly influence the generation of DAMPs and vice versa. (2) These signals initiate the chemotaxis and activation of immune cells such as other neutrophils that can sense DAMPs in several ways, including recognition by pattern recognition receptors, leading to inflammasome activation. Inflammasome signaling propagates a proinflammatory response through the increased release of proinflammatory cytokines (e.g., IL-1β, CXCL1) and acute phase proteins (e.g., α1AGP, CRP), as well as mobilization of peripheral immune cells. (3) The presentation of clozapine neoantigens in the context of specific HLA haplotypes on antigen-presenting cells are detected by T cells expressing cognate T cell receptors. In the presence of appropriate co-stimulatory signals, an adaptive immune response is initiated that, if unresolved through tolerance, leads to the targeted destruction of granulocyte precursors and the onset of IDIAG. The inhibition of myeloperoxidase with PF-1355 significantly attenuates clozapine-induced inflammation, ultimately reducing the chance of adaptive immune activation and progression to IDIAG.

Moreover, this work provided compelling support for the requisite of myeloperoxidase activation during clozapine-mediated inflammation. Myeloperoxidase inhibition, using the highly effective myeloperoxidase inhibitor PF-1355 (Figure 3), dampened the clozapine-induced release of the DAMP and inflammasome product IL-1β along with other inflammatory mediators in PMA-differentiated THP-1 macrophages (Figure 4). In our rat model, pretreatment with PF-1355 attenuated clozapine-mediated increases in neutrophil chemotaxis and related serum inflammatory mediators (Figure 5), by preventing the mobilization of mature neutrophils from the bone marrow to the blood and spleen (Figure 6), and the release of DAMPs and other molecules from organs including the liver, spleen, and bone marrow (Figure 7). Taking these data together, we arrive at our updated model to describe how clozapine induces an immune response that, in some patients, may ultimately lead to the onset of IDIAG (Figure 8).

Our model postulates that the immune response to clozapine is triggered following detection of the cellular damage and stress caused by the binding of myeloperoxidase-generated reactive metabolites. However, myeloperoxidase does not account for the generation of all clozapine-reactive metabolites in neutrophils, since myeloperoxidase knockout neutrophils still exhibit detectable covalent binding when treated with clozapine ex vivo [27], nor does covalent binding alone result in the onset of IDIAG, since covalent binding has been detected in the neutrophils of patients chronically treated with clozapine who did not develop IDIAG [32]. Nevertheless, the early proinflammatory response to clozapine is a requisite for any potential progression to an adaptive immune response that culminates in IDIAG, and the current study suggests that the DAMPs, specifically those generated by myeloperoxidase during clozapine treatment, are sufficient to trigger the release of other proinflammatory mediators and induce a robust yet transient proinflammatory response. Thus, inhibition of myeloperoxidase may be considered a future strategy to reduce the risk of progression to IDIAG during clozapine therapy.

However, the potential implications of this work extend well beyond the use of clozapine. Many drugs associated with IDIAG and neutropenia, particularly those containing electron-rich functional groups that contain nitrogen or sulfur, are oxidized by activated neutrophils, other myeloid cells, enzyme systems including myeloperoxidase, or simply by hypochlorous acid [29]. Primary aromatic amines (e.g., the antiarrhythmic procainamide [71,72]), other nitrogen-containing compounds (e.g., the antimalarial amodiaquine [27,73]), sulfur-containing compounds (e.g., the antithyroid methimazole (thiamazole) [74]), and countless other xenobiotics have been shown to undergo myeloperoxidase-mediated oxidation to products including hydroxylamines, nitroso metabolites, and chloramines [29]. Moreover, since reactive metabolites typically bind to proteins close to the site of generation [75], the neoantigens and cell stress caused by myeloperoxidase drug bioactivation may play a role in the onset of other idiosyncratic blood-related dyscrasias, such as hemolytic anemia, thrombocytopenia, or even vasculitis. Thus, acute inhibition of myeloperoxidase in patients during the initiation of IDIAG- (and other relevant IDR-) associated drugs may represent a strategy to prevent early immune activation and promote tolerance, ultimately avoiding progression to a severe IDR.

Beyond our work, inhibition of myeloperoxidase has been proposed as a treatment for a number of conditions in which there is compelling evidence that myeloperoxidase and/or its oxidant products are central to disease etiology (extensively reviewed in [76,77]). The oxidative stress and resulting proinflammatory milieu observed in conditions such as atherosclerosis [78], rheumatoid arthritis [79], kidney disease [80], inflammatory bowel disease [81], and neurodegenerative disorders [82] have commonly been attributed to the activity or overactivity of myeloperoxidase. In addition to the work characterizing PF-1355 [35,36], hundreds of preclinical studies have investigated the effects of a number of structurally-diverse myeloperoxidase inhibitors, including 4-aminobenzohydrazide, other hydrazines and hydrazides, and peptide-based inhibitors, as well as derivatives of macrocyclic triazolopyridine, ferulic acid, indole alkylamine, thioxanthine, and thiopyrimidinone [83]. Although many of these studies have demonstrated positive results in reducing inflammation or disease severity, many of the inhibitors used come with unacceptable side effect profiles (particularly the hydrazides [50]) and are relegated to mechanistic in vitro and in vivo experiments. Of the handful of inhibitors (AZD5904, AZD3241, and AZD4831 from AstraZeneca and PF06282999 from Pfizer) that have progressed to clinical trials, only one (AZD4831) is still under investigation, with the others dropped due to lack of efficacy or intolerable adverse effects [83]. Undoubtedly, further research is necessary to develop a myeloperoxidase inhibitor that can be safely given to patients; however, this approach may hold promise in the prevention of IDIAG.

In our working model, we hypothesize that myeloperoxidase triggers inflammation directly through the detection of cell stress and DAMP release caused by the myeloperoxidase-mediated bioactivation of clozapine. Myeloperoxidase is implicated in numerous disease states that are not drug-induced, and it is possible that other actions of myeloperoxidase are also important. For instance, myeloperoxidase, either directly or through the generation of hypochlorous acid, can oxidize various endogenous biomolecules, including amino acids, RNA, DNA, and lipoproteins [84,85,86,87]. Thus, clozapine-induced increases in myeloperoxidase activity could result in oxidative damage to host tissue, DAMP generation, and a contribution to the inflammatory response [88]. Additionally, myeloperoxidase is a key component of neutrophil [89], macrophage [90], and other extracellular traps, which are networks of chromatin, granule proteins, and other intracellular components that can be released from cells during infection and sterile inflammation [91]. Clozapine may trigger extracellular trap release, and it could be the extracellular myeloperoxidase released in these traps that contributes to the inflammation observed. One in vitro study indicated that human neutrophils treated with clozapine did not exhibit significant extracellular trap formation [92]. However, the concentration of clozapine tested (0.8 μM) was much too low to have therapeutic relevance.

Ultimately, the results of this study reaffirm the potential clinical translatability of our clozapine rat model and highlight the requisite of myeloperoxidase in the induction of clozapine-mediated inflammation. The demonstration of an inflammatory response in rats at exposure levels that represent those of new clozapine patients supports further examination of the peripheral immune response in human participants. While the exact pathway(s) through which myeloperoxidase triggers clozapine-induced inflammation remains to be determined, these studies enhance our understanding of the mechanistic basis of IDIAG. If a reactive drug or metabolite cannot induce an inflammatory immune response, it cannot trigger an adaptive immune response that, in some patients, leads to an IDR [19]. With clozapine, myeloperoxidase is responsible for promoting a proinflammatory response, accompanied by leukocyte chemotaxis and mediator release, and myeloperoxidase inhibition significantly attenuates this response. Overall, characterization of these early immune steps and identification of unique biomarkers could be used to screen drug candidates for the potential to cause serious IDRs, which would facilitate the development of safer drugs.

## 4. Materials and Methods

### 4.1. Chemicals and Reagents

Clozapine was donated by Apotex (Toronto, ON, Canada), and olanzapine was purchased from Toronto Research Chemicals Inc. (Toronto, ON, Canada). Fluperlapine was purchased from MedChemExpress (Princeton, NJ, USA). The mechanism-based myeloperoxidase inhibitor PF-1355 [2-(6-(2,5-dimethoxyphenyl)-4-oxo-2-thioxo-3,4-dihydropyrimidin-1(2H)-yl)acetamide] was kindly provided by Pfizer (New York, NY, USA). Qualified One Shot™ fetal bovine serum (FBS; Gibco, Grand Island, NY, USA) was heat-inactivated at 56 °C for 30 min in-house, as necessary. PMA was purchased from BioShop Canada Inc. (Burlington, ON, Canada). Flow cytometry reagents were obtained from BD Biosciences (Mississauga, ON, Canada), unless otherwise specified. All other reagents were commercially obtained.

### 4.2. Cell Culture, Maintenance, and Drug Exposure

THP-1 monocytes (TIB-202) were obtained from the American Type Culture Collection (ATCC, Manassas, VA, USA) and maintained at 37 °C, 5% CO_2_ in filter-sterilized ATCC high glucose RPMI-1640 media, supplemented with 10% heat-inactivated FBS. For each experiment, THP-1 monocytes (4 × 10^5^ cells/mL; passage 3–20) were stimulated with PMA (25 ng/mL) for 72 h to allow for macrophage differentiation. Cells were washed with D-PBS and incubated in fresh media for 24 h prior to drug exposure in fresh media. Macrophages were treated with clozapine, fluperlapine, or olanzapine (10 μg/mL; 30 μM), the irreversible myeloperoxidase inhibitor PF-1355 (0.01–10 μg/mL; 3 –30 μM), or vehicle control media (0.1% dimethyl sulfoxide (DMSO)). Clozapine (10 μg/mL) was also coincubated with PF-1355 (0.01–10 μg/mL), following a 10 min pre-incubation with PF-1355 to inhibit myeloperoxidase activity. Cells were exposed to the drug for 1–24 h, following which conditioned media and cell lysates were collected for analysis. Cells were lysed with radioimmunoprecipitation assay (RIPA) buffer (Abcam, Waltham, MA, USA), supplemented with a Protease and Phosphatase Inhibitor Cocktail (Abcam, Waltham, MA, USA).

### 4.3. Verification of Myeloperoxidase Protein Expression in THP-1 Cells

Protein (10 μg) from THP-1 monocytes or PMA-differentiated macrophages was combined with sample reducing buffer, denatured, and run on a 10% Mini-PROTEAN TGX Stain-Free precast gel (Bio-Rad Laboratories, Hercules, CA, USA), prior to transfer to a 0.45 μm nitrocellulose membrane. Membranes were stained for total protein using a Novex™ Reversible Nitrocellulose Membrane Protein Stain Kit (Thermo Fisher, Waltham, MA, USA), blocked, incubated overnight with a rabbit polyclonal myeloperoxidase antibody (1:2000 dilution; Abcam, Waltham, MA, USA), incubated with a goat anti-rabbit horseradish peroxidase secondary antibody (1:20,000 dilution; Sigma-Aldrich, St. Louis, MO, USA), and then visualized with chemiluminescent detection on a ChemiDoc imager (Bio-Rad Laboratories, Hercules, CA, USA). Densitometry was performed using Image Studio™ Lite software (version 5.2; LI-COR Biosciences, Lincoln, NE, USA).

### 4.4. Assessment of THP-1 Macrophage Viability

Cell viability was assessed at each timepoint using a CCK-8 (WST-1) cell proliferation assay (GLPBio, Montclair, CA, USA), according to the manufacturer’s instructions. 10% DMSO was employed as a positive control for cell death [93].

### 4.5. Measurement of Myeloperoxidase Activity

Peroxidation activity in THP-1 macrophage and rat neutrophil lysates was measured using an EnzCheck™ Myeloperoxidase Activity Assay Kit (Invitrogen, Waltham, MA, USA). Recombinant human myeloperoxidase (1.5–200 ng; Sigma-Aldrich, Oakville, ON, Canada) was used to generate a standard curve and determine unknown peroxidase levels.

### 4.6. Animal Treatment

Female Sprague Dawley rats (200–250 g) were obtained from Charles River (St. Constant, QC, USA) and double- or triple-housed with a 12/12 h light/dark cycle at 22 °C. Prior to experiments, animals were acclimatized and handled daily for >1 week and provided access to rodent chow (Harlen Teklad, Madison, WI, USA) and water ad libitum. All animal protocols were approved by the University of Toronto Animal Care Committee. Clozapine (2.5, 5, 10, or 30 mg/kg) or saline vehicle was prepared as previously described [22] and was administered between 8 and 9 am via intraperitoneal (IP) injection. Immune parameters were evaluated from 0–24 h post-dose. To analyze the effect of myeloperoxidase inhibition, rats either received a single predose of PF-1355 (100 mg/kg) or vehicle (0.5% hydroxypropyl methylcellulose) via oral gavage 1 h prior to a single dose of clozapine (30 mg/kg).

### 4.7. Measurement of Serum Clozapine Concentrations

**Figure 9:**
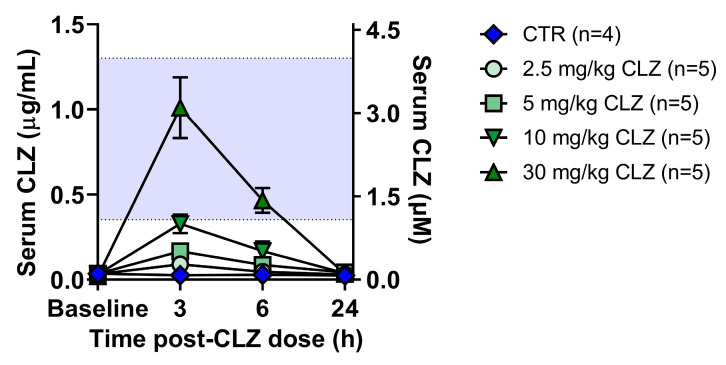
Measurement of clozapine blood levels in rats by LC-MS/MS. Serum drug concentrations in female Sprague Dawley rats over 24 h following treatment with vehicle (control) or clozapine (2.5, 5, 10, or 30 mg/kg, IP). Drug concentrations were measured using a TSQ Altis Triple Quadrupole Mass Spectrometer interfaced with a 1260 Infinity II Prime LC System and analyzed using XCalibur software. Results are presented as the mean ± SD, where the purple shaded area represents the average therapeutic range reported in patients [94]. CTR, control; CLZ, clozapine.

Serum was isolated from procoagulated whole blood, collected over 24 h from the tail vein or via cardiac puncture (endpoint only), by centrifugation at 10,000× *g* for 10 min at 4 °C. Clozapine serum samples were combined with methanol and internal standard carbamazepine (0.5 μg/mL, 1.5 μM), Clozapine standards were prepared in methanol (0.01−2.0 μg/mL, 0.03 μM−6.1 μM), combined with internal standard carbamazepine (0.5 μg/mL, 1.5 μM), and blank rat serum. Serum proteins were precipitated for >1 h at −20 °C, and supernatants were isolated following centrifugation at 16,000× *g* for 10 min. Clozapine drug levels were measured using a TSQ Altis Triple Quadrupole Mass Spectrometer (Thermo Fisher, Waltham, MA, USA) interfaced with a 1260 Infinity II Prime LC System (Agilent Technologies, Santa Clara, CA, USA) and analyzed using XCalibur software (Thermo Fisher, Waltham, MA, USA). The mobile phase consisted of 60% aqueous (0.1% formic acid, 1 mM ammonium formate in H_2_O) and 40% organic (acetonitrile), and a Poroshell 120 EC-C18, 3.0 × 50 mm, 2.7 μm column (Agilent Technologies, Santa Clara, CA, USA) was employed. Quantification was performed using XCalibur software (Thermo Fisher, Waltham, MA, USA) (Figure 9).

### 4.8. Tissue Processing and Neutrophil Enrichment

EDTA-anticoagulated whole blood was collected for automated differential blood counts using a VetScan HM5 (Union City, CA), with samples run in duplicate within an hour of collection. Liver, spleen, and bone marrow were collected and homogenized using ice-cold Dounce homogenizers and RIPA buffer, supplemented with a Protease and Phosphatase Inhibitor Cocktail and, along with serum, were collected for measurement of inflammatory mediators. Blood, spleen, and bone marrow were also collected for leukocyte phenotyping via flow cytometry and for neutrophil enrichment to measure myeloperoxidase activity. Neutrophils were isolated from single cell suspensions using a custom rat magnetic bead negative selection kit (StemCell Technologies Inc., Vancouver, BC). 1–2 × 10^8^ cells were incubated with an isolation antibody cocktail prior to the addition of dextran magnetic beads and incubation in the presence of a strong magnetic field to remove non-neutrophil cellular populations. Enriched neutrophils were then lysed as above.

### 4.9. Measurement of Inflammatory Cytokines and Mediators

The following ELISA kits were used for THP-1 cell culture supernatants: IL-1β, CXCL1, and CRP (R&D Systems, Minneapolis, MN, USA). The following ELISA kits were used for rat tissue: IL-1β, TNF-α, CXCL1, α-1-acid glycoprotein (α1AGP), and calprotectin (S100A8/A9 heterodimer; R&D Systems, Minneapolis, MN, USA), and corticosterone (Invitrogen, Waltham, MA, USA). 

### 4.10. Flow Cytometry

Flow cytometric phenotyping of basic leukocyte populations in whole blood, spleen, and bone marrow was conducted as described in [60], with slight modifications to assess CD62L expression on granulocytes [20,95]. Samples were maintained at 4 °C unless otherwise noted. Briefly, single-cell suspensions of bone marrow and spleen were prepared in 1% FBS in PBS (FACS) buffer by straining whole tissue through 70 μm strainers. EDTA-anticoagulated whole blood was lysed in 1× BD Pharm Lyse™ buffer to remove erythrocytes, and remaining immune cells were resuspended in FACS buffer. Viable cells were counted using Trypan blue staining solution (Gibco, Grand Island, NY, USA) on a hemocytometer, and 1 × 10^6^ cells/sample were plated and blocked with anti-CD32 to prevent Fc-mediated non-specific binding prior to staining (see Table 1 for antibody details). Following extracellular and viability staining, cells were fixed and permeabilized using the Foxp3/Transcription Factor Staining Buffer Set (eBioscience, San Diego, CA, USA) to preserve the integrity of antigens during intracellular CD68 staining. Flow cytometric compensation was performed using species-appropriate BD CompBeads, and cells were analyzed using a multi-parameter flow cytometer (BD LSRFortessa X-20 Cell Analyzer). For the identification of positive and negative populations, the fluorescence minus one (FMO) approach was utilized to adjust for background antibody fluorescence. The data were then analyzed using FlowJo software (version 10.0.7). The gating strategy is presented in Supplementary Materials, Figure S4.

### 4.11. Statistical Analyses

Data are expressed as the mean ± SD and were analyzed using GraphPad Prism software version 8.4.3 (GraphPad, San Diego, CA, USA). Differences between groups were evaluated using Student’s t test or one- or two-way ANOVA with the Holm-Sidak multiple comparison test, whereby an adjusted *p* value < 0.05 was considered statistically significant.

## Figures and Tables

**Table 1:** Monoclonal antibodies and dyes used for flow cytometric leukocyte phenotyping.

Laser	Filter	Marker	Clone	Fluorochrome	Supplier
-	-	CD32 *	D34-485	-	BD Pharmingen
Violet (407 nm)	780/60	CD8a	OX-8	BV786	BD Biosciences
	710/50	CD172a	OX-41	BV711	
	670/30	CD161	10/78	BV650	
	610/20	CD45RA	OX-33	BV605	
	525/50	Live/Dead	-	e506	eBioscience
	450/50	CD62L	HLR1	BV421	BD Biosciences
Blue (488 nm)	710/50	CD3e	G4.18	PerCP-eFluor710	eBioscience
	530/30	His48	His48	FITC	BD Pharmingen
Yellow-Green (561 nm)	780/60	CD43	W3/13	PE-Cy7	BioLegend
	610/20	CD68 **	ED1	Dylight594	Novus Biologicals
Red (640 nm)	780/60	CD45	OX-1	APC-Cy7	BD Biosciences
	670/30	CD11b	WT.5	APC	
Ultraviolet (355 nm)	515/30	RT1B (MHCII)	OX-6	BUV496	BD Biosciences
	379/28	CD4	OX-35	BUV395	

* CD32 used to block Fc-mediated non-specific binding. ** CD68 used in intracellular staining.

## Data Availability

The data presented in this study are available by request from the corresponding author.

## Supplementary Materials

The following supporting information can be downloaded at: https://www.mdpi.com/article/10.3390/ijms24021243/s1, Figure S1: Clozapine triggers dose-related, organ-specific changes in inflammatory mediators and DAMPs in rats at 24 h; Figure S2: Clozapine triggers dose-related decreases in the organ weights of rats at 24 h; Figure S3: Myeloperoxidase is expressed in THP-1 cells; Figure S4: Gating strategy for flow cytometry panel.
