# Supplementary material for: The Role of Myeloperoxidase in Clozapine-Induced Inflammation: A Mechanistic Update for Idiosyncratic Drug-Induced Agranulocytosis
Source: Int J Mol Sci. 2023 Jan 8;24(2):1243. doi: 10.3390/ijms24021243 (PMC9862306; doi:10.3390/ijms24021243)
Supplement: Supplementary file 1 [file ijms-24-01243-s001.zip › ijms-2118777-supplementary.pdf]

International Journal of Molecular Sciences

The role of myeloperoxidase in clozapine-induced inflammation: a mechanistic update for idiosyncratic drug-induced agranulocytosis

Samantha Christine Sernoskie, Alison Jee, and Jack Uetrecht

Supplementary Materials

1.1. Supplemental Figure S1

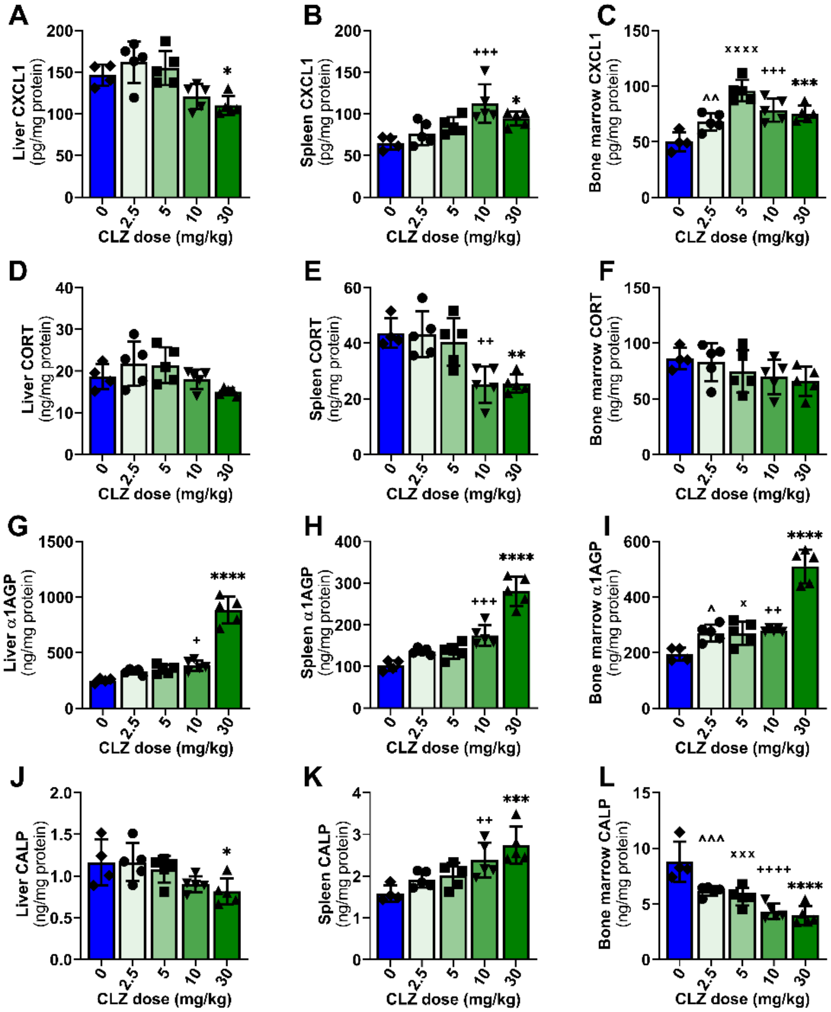

Supplemental Figure S1. Clozapine triggers dose-related, organ-specific changes in inflammatory mediators and DAMPs in rats at 24 h. Concentrations of CXCL1 (A-C), corticosterone (D-F), α1AGP (G-I), and calprotectin (J-L) in the liver (A,D,G,J), spleen (B,E,H,K), and bone marrow (C,F,I,J) in female Sprague-Dawley rats at 24 hours following treatment with vehicle (control), or clozapine (2.5, 5, 10, or 30 mg/kg, IP). Results are presented as the mean ± SD and statistical difference between groups was determined by one-way ANOVA with the Holm-Sidak's test for multiple comparisons. Inflammatory mediators were quantified using commercially available ELISA kits. CTR, control; CLZ, clozapine; CORT, corticosterone; CALP, calprotectin; ^, $p<0.05$; ^^, $p<0.01$; ^^^, $p<0.001$; (2.5 mg/kg clozapine vs. control); x, $p<0.05$; xxx, $p<0.001$; xxxx, $p<0.0001$ (5 mg/kg clozapine vs. control); +, $p<0.05$; ++, $p<0.01$; +++, $p<0.001$; ++++, $p<0.0001$ (10 mg/kg clozapine vs. control); *, $p<0.05$; **, $p<0.01$; ***, $p<0.001$; ****, $p<0.0001$ (30 mg/kg clozapine vs. control). n=4-5/group.

1.2. Supplemental Figure S2

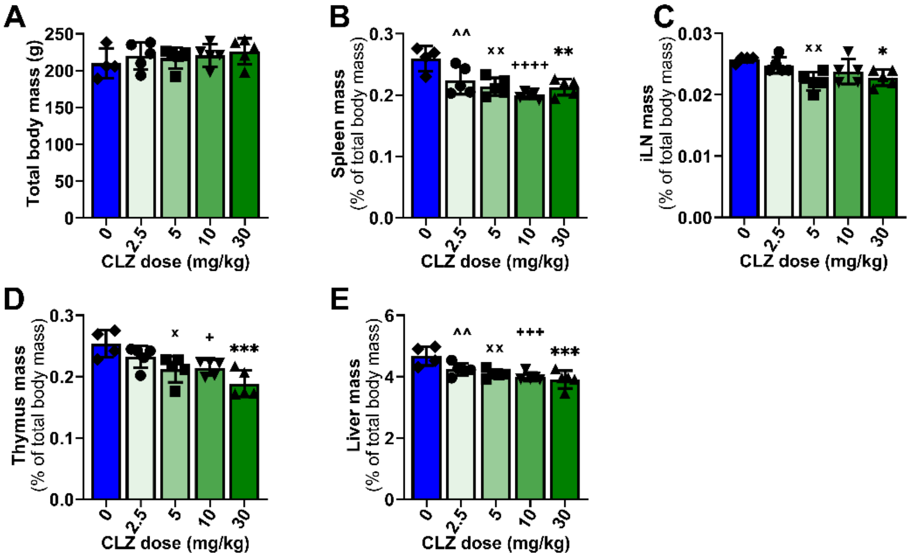

Supplemental Figure S2. Clozapine triggers dose-related decreases in the organ weights of rats at 24 h. (A) Total body mass and (B-E) normalized organ masses of spleen (B), inguinal lymph nodes (C), thymus (D), and liver (E), presented as a percentage of total body mass, in female Sprague-Dawley rats at 24 hours following treatment with vehicle (control), or clozapine (2.5, 5, 10, or 30 mg/kg, IP). Results are presented as the mean ± SD and statistical difference between groups was determined by one-way ANOVA with the Holm-Sidak's test for multiple comparisons. CTR, control; CLZ, clozapine; iLN, inguinal lymph nodes; ^^, p<0.01; (2.5 mg/kg clozapine vs. con-trol); x, p<0.05; xx, p<0.01; (5 mg/kg clozapine vs. control); +, p<0.05; +++, p<0.001; ++++, p<0.0001 (10 mg/kg clozapine vs. control); *, p<0.05; **, p<0.01; ***, p<0.001; (30 mg/kg clozapine vs. control). n=4-5/group.

1.3. Supplemental Figure S3

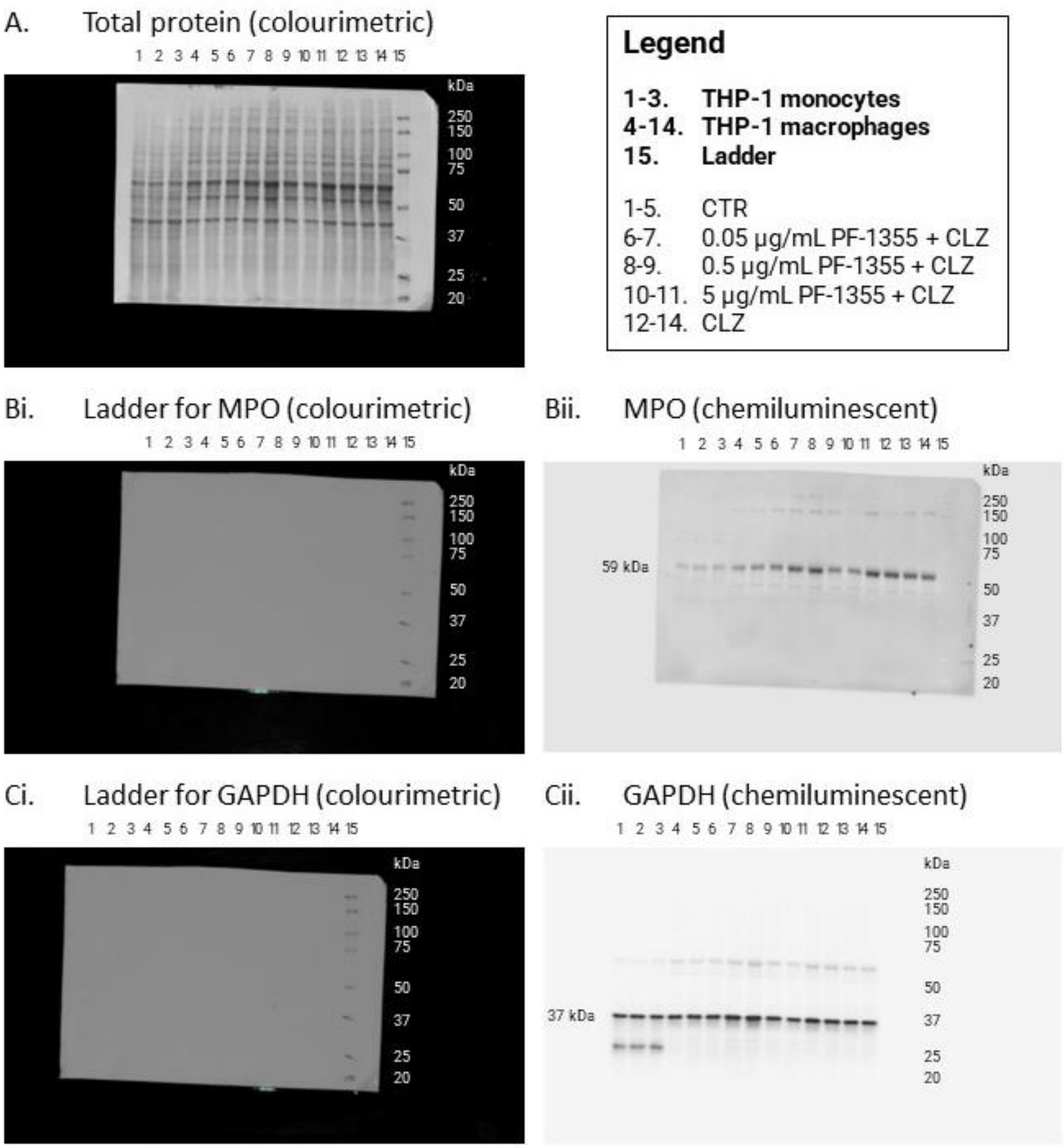

Supplemental Figure S3. Myeloperoxidase is expressed in THP-1 cells. THP-1 macrophages, differentiated with 25 ng/mL of PMA for 72 h, were incubated with 0.1% DMSO (control), clozapine (10 µg/mL) ± PF-1355 (0.05, 0.5, or 5 µg/mL) or undifferentiated THP-1 monocytes were incubated with 0.1% DMSO for 24 hours. Conditioned media was removed, and cells were lysed using radioimmunoprecipitation assay buffer (RIPA) buffer (Abcam, Toronto, ON), supplemented with Protease and Phosphatase Inhibitor Cocktail (Abcam, Toronto, ON). Protein (10 µg) was combined with sample reducing buffer, denatured, and run on a 10% Mini-PROTEAN TGX Stain-Free precast gel (Bio-Rad Laboratories, Hercules, CA), prior to transfer to a 0.45 µm nitrocellulose membrane. Membranes were stained for total protein using a Novex™ Reversible Nitrocellulose Membrane Protein Stain Kit (Thermo Fisher, Waltham, MA), blocked, incubated overnight with a rabbit polyclonal myeloperoxidase antibody (1:2,000 dilution; Abcam, Waltham, MA), incubated with a goat anti-rabbit horseradish peroxidase secondary antibody (1:20,000 dilution; Sigma-Aldrich, St. Louis, MO), and then visualized with chemiluminescent detection on a ChemiDoc imager (Bio-Rad Laboratories, Hercules, CA). Densitometry was performed using Image Studio™ Lite software (version 5.2; LI-COR Biosciences, Lincoln, NE). Membranes were then stripped, reblocked, and stained for glyceraldehyde 3-phosphate dehydrogenase (GAPDH; 1:20,000 dilution; Thermo Fisher, Waltham, MA), although total protein staining was used for myeloperoxidase band density normalization. (A-C) Unadjusted images used for band density normalization in Figure

3 of the main text. (A) Colourimetric image for total protein staining (0.001 second exposure). (B) Immunoblot analysis to detect myeloperoxidase protein expression in THP-1 cells, separated into (Bi) colourimetric image for ladder (0.001 second exposure) an (Bii) chemiluminescent image for myeloperoxidase staining (30 second exposure). (C) Immunoblot analysis to detect GAPDH protein expression in THP-1 cells, separated into (Ci) colourimetric image for ladder (0.001 second exposure) and (Cii) chemiluminescent image for GAPDH staining (30 second exposure).

1.4. Supplemental Figure S4

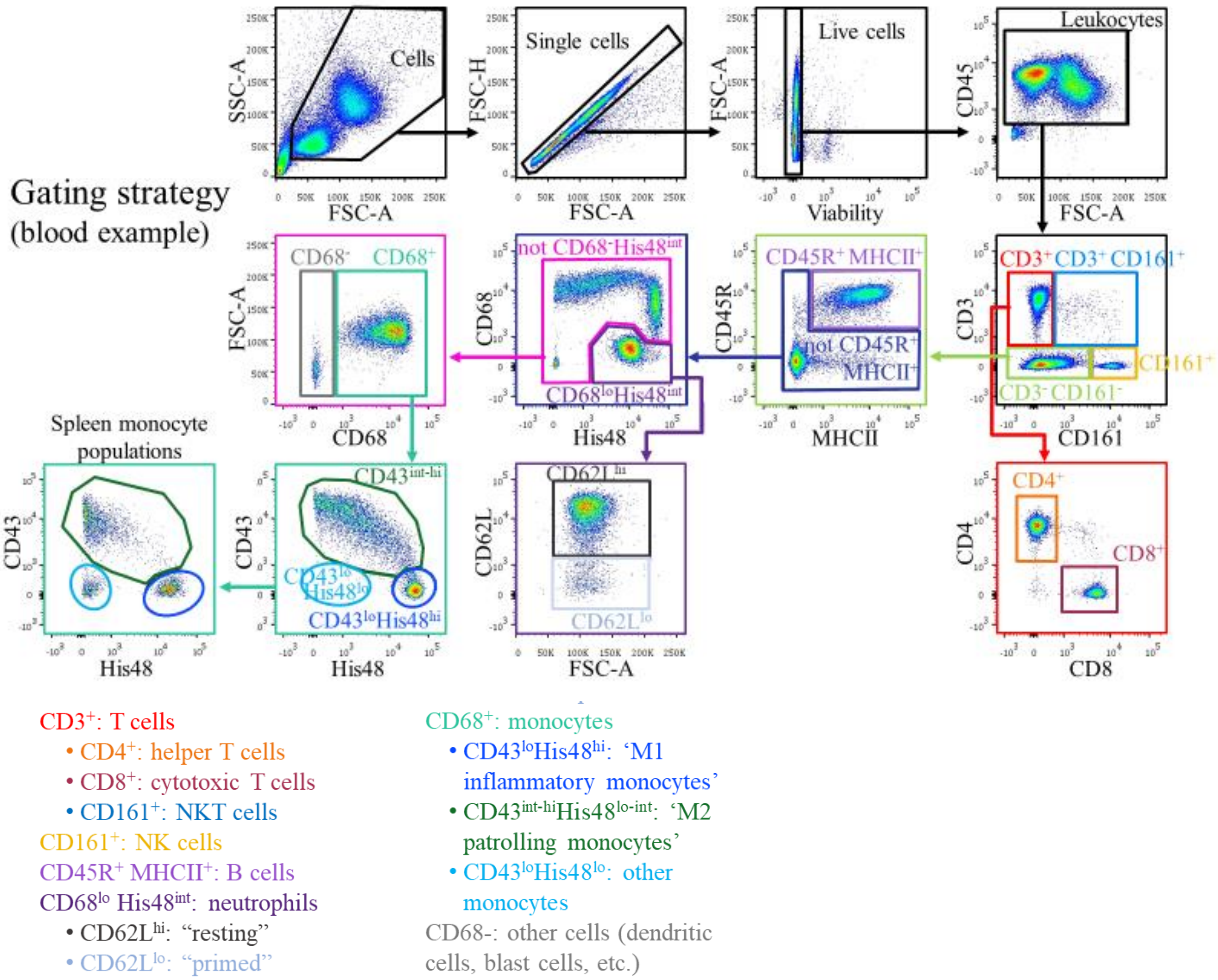

Supplemental Figure S4. Gating strategy for flow cytometry panel. See legend above for classification of cells based on markers. Gating strategy, shown using blood sample, adapted from [60] Barnett-Vanes, A.; Sharrock, A.; Birrell, M.A.; Rankin, S. A Single 9-Colour Flow Cytometric Method to Characterise Major Leukocyte Populations in the Rat: Validation in a Model of LPS-Induced Pulmonary Inflammation. *PLoS One* **2016**, *11*, e0142520, doi:10.1371/journal.pone.0142520.
